# Metrnl ameliorates myocardial ischemia–reperfusion injury by activating AMPK-mediated M2 macrophage polarization

**DOI:** 10.1186/s10020-025-01150-4

**Published:** 2025-03-13

**Authors:** De-Xin Chen, Yang-Yi Feng, Hai-Yan Wang, Chuang-Hong Lu, De-Zhao Liu, Chen Gong, Yan Xue, Na Na, Feng Huang

**Affiliations:** 1 Department of Cardiology, Guangxi Key Laboratory of Precision Medicine in Cardio-Cerebrovascular Diseases Control and Prevention, Guangxi Clinical Research Center for Cardio-Cerebrovascular Diseases, The First Affiliated Hospital of Guangxi Medical University, No.6 Shuangyong Road, Nanning, 530021 Guangxi China; 2 Department of Neuroscience, Scripps Research Institute, No.10550 North Torrey Pines Road, La Jolla, CA 92037 USA

**Keywords:** Metrnl, AMPK, Macrophage polarization, Inflammation, Apoptosis, Myocardial ischemia–reperfusion injury

## Abstract

**Background:**

Meteorin-like hormone (Metrnl) is prominently expressed in activated M2 macrophages and has demonstrated potential therapeutic effects in a range of cardiovascular diseases by modulating inflammatory responses. Nevertheless, its precise role and the underlying mechanisms in myocardial ischemia/reperfusion injury (MI/RI) are not fully understood. This study examined whether Metrnl can mitigate MI/RI through the AMPK-mediated polarization of M2 macrophages.

**Methods:**

In vivo, adeno-associated virus 9 containing the F4/80 promoter (AAV9-F4/80) was utilized to overexpress Metrnl in mouse cardiac macrophages before MI/RI surgery. In vitro, mouse bone marrow-derived macrophages (BMDMs) were treated with recombinant protein Metrnl, and the human cardiomyocyte cell line AC16 was subjected to hypoxia/reoxygenation (H/R) after co-culture with the supernatant of these macrophages. Cardiac function was assessed via echocardiography, H&E staining, and Evans blue-TTC staining. Inflammatory infiltration was evaluated by RT-qPCR and ELISA, apoptosis by Western blotting and TUNEL staining, and macrophage polarization by immunofluorescence staining and flow cytometry.

**Results:**

In vivo, Metrnl overexpression in cardiac macrophages significantly attenuated MI/RI, as evidenced by reduced myocardial infarct size, enhancement of cardiac function, diminished inflammatory cell infiltration, and decreased cardiomyocyte apoptosis. Furthermore, Metrnl overexpression promoted M1 to M2 macrophage polarization. In vitro, BMDMs treated with Metrnl shifted towards M2 polarization, characterized by decreased expression of inflammatory cytokines (IL-1β, MCP-1, TNF-α) and increased expression of the anti-inflammatory cytokine IL-10. Additionally, supernatant from Metrnl-treated macrophages protected AC16 cells from apoptosis under H/R conditions, as evidenced by decreased BAX expression and increased BCL-2 expression. However, these effects of Metrnl were inhibited by the AMPK inhibitor Compound C.

**Conclusions:**

Metrnl alleviates MI/RI by activating AMPK-mediated M2 macrophage polarization to attenuate inflammatory response and cardiomyocyte apoptosis. This study highlights the therapeutic potential of Metrnl in MI/RI, and identifies it as a promising target for the treatment of ischemic heart disease.

**Supplementary Information:**

The online version contains supplementary material available at 10.1186/s10020-025-01150-4.

## Introduction

The persistence of high incidence and mortality rates of acute myocardial infarction (AMI), driven by various factors including high-salt and high-fat diets, increased work stress, and decreased physical activity, poses a substantial health and socioeconomic burden (Rawal et al. 2023). Clinically, AMI is primarily managed through reperfusion therapy via pharmacologic thrombolysis or percutaneous coronary intervention (PCI). However, the restoration of coronary blood flow during reperfusion often precipitates myocardial ischemia/reperfusion injury (MI/RI), characterized by life-threatening complications such as malignant arrhythmias, the no-reflow phenomenon, myocardial stunning, and exacerbated myocardial necrosis, potentially culminating in patient mortality (Algoet et al. 2023). Effective clinical strategies for treating or preventing MI/RI remain elusive, necessitating a deeper understanding of its underlying mechanisms, the identification of key therapeutic targets, and the development of effective interventions to prevent or ameliorate this injurious process.

MI/RI is prominently characterized by inflammatory damage and cardiomyocyte apoptosis, with the macrophage-mediated inflammatory response as a pivotal factor (Huang et al. 2020). Macrophages are classified into two types based on their phenotype and cytokine secretion: M1 macrophages, exhibiting pro-inflammatory and deleterious effects, and M2 macrophages, demonstrating anti-inflammatory and reparative functionalities (Liu et al. 2021). Previous studies have shown that modulating macrophage polarization by decreasing M1 polarization or enhancing M2 polarization can ameliorate MI/RI (Ge et al. 2021; Gao et al. 2023). Notably, Meteorin-like hormone (Metrnl), a protein highly expressed in activated M2 macrophages and in both human and rodent hearts, plays a crucial role in regulating energy metabolism, cold exposure responses, inflammation, and obesity (Ushach et al. 2015). Metrnl is closely associated with energy metabolism and may regulate macrophage polarization through the AMPK pathway.

AMPK regulates cellular energy metabolism and biosynthesis by monitoring the intracellular AMP/ATP ratio, which has a crucial impact on the polarization state of macrophages. Previous studies have demonstrated that Metrnl attenuates lipopolysaccharide (LPS)-induced inflammation via the AMPK pathway (Jung et al. 2018) and promotes macrophage polarization toward the M2 type (Song et al. 2023). Furthermore, Metrnl assumes a significant role in cardiovascular diseases, such as improving diabetic cardiomyopathy, promoting post-infarction angiogenesis, and delaying ventricular remodeling after heart failure (Reboll et al. 2022; Lu et al. 2023a; Rupérez, et al. 2021). However, research on the protective role of Metrnl in MI/RI and its underlying mechanisms is still in the exploratory stage. Given Metrnl's potent cardioprotective properties, its capacity to alleviate inflammation through AMPK activation, and its potential to induce M2 macrophage polarization, we hypothesized that Metrnl could attenuate MI/RI by activating AMPK-induced polarization of M2 macrophages, thereby reducing the inflammatory response and cardiomyocyte apoptosis.

The present study demonstrated that the expression of Metrnl was downregulated in macrophages in the infarct zone of the heart after MI/RI. Specific overexpression of Metrnl in cardiac macrophages, achieved through an adeno-associated virus, effectively inhibited M1 macrophage polarization, promoted M2 macrophage polarization, attenuated the inflammatory response and cardiomyocyte apoptosis, and ameliorated MI/RI. Consistent with the findings of the in vivo experiments, in vitro studies further confirmed that Metrnl promotes the polarization of macrophages from M1 to M2 and decreases the release of inflammatory factors through AMPK activation, thus attenuating the effects of hypoxia/reoxygenation (H/R)-induced myocardial apoptotic injury. This study further elucidates the therapeutic role of Metrnl in MI/RI and suggests that Metrnl may be an effective therapeutic target for ischemic heart disease.

## Materials and methods

### Experimental animals

C57BL/6 male mice, weighing 22–25 g, were procured from Guangxi Medical University Laboratory Animal Center (Nanning, China). Mice were housed in a controlled environment with a light/dark cycle chamber, maintained at a temperature range of 20–25 °C, and provided with standard food and water ad libitum. One week before surgery or intervention, mice were acclimatized to relieve environmental stress. Animal experiments in this study were approved by the Animal Care and Welfare Committee of Guangxi Medical University (202,111,022).

### Cell culture

Bone marrow-derived macrophages (BMDMs) were isolated from mouse femoral and tibial bone marrow sterilized with 75% ethanol, rinsed with PBS (containing 1% fetal bovine serum and 2 mM EDTA), and filtered through a 70 um filter. Bone marrow erythrocytes were lysed with ACK lysis buffer (Gibco) for 5 min. The resulting cells were centrifuged for 5 min (4 °C, 1000 rpm/min) and then resuspended with IMDM (containing 10% fetal bovine serum and 20 ng/mL M-CSF [R&D Systems]), and then cells were seeded in plates and cultured for 5 days to obtain macrophages (Huang et al. 2021). Human Cardiomyocyte Cell Line AC16 (Procell, Wuhan, China) were cultured in DMEM medium with 10% fetal bovine serum (Gibco, New York, USA).

### MI/RI model establishment

The myocardial ischemia–reperfusion injury(MI/RI) model is based on our group's previous research (He 2023). Mice were anesthetized via intraperitoneal injection with 1.25% avertin (Aibei Biotechnology, Nanjing, China) at a dosage of 0.3 ml per 20 g body weight, and then connected to a MiniVent 845 ventilator (model 845, Harvard Apparatus, Germany). The edge of the pectoralis major muscle was obliquely incised, and the pectoralis minor muscle was directly dissected to expose the heart through the left fourth intercostal space. The left anterior descending coronary artery (LAD) was ligated with a No. 7 − 0 silk suture, indicated by an obvious color change of the left anterior myocardium from red to white and a significant reduction or cessation of ventricular wall motion. The ligation was released after 30 min, and tissue samples were collected 24 h after reperfusion. For the Sham group, LAD ligation was not performed.

### In vivo genetic intervention and grouping

Adeno-associated virus serotype 9 vector with the F4/80 promoter (AAV9-F4/80, Hambio, Shanghai, China) served as a reagent carrier. AAV9-F4/80 containing the mouse Metrnl (NM_144797.3) enhancement sequence (AAV9-F4/80-Metrnl-ZsGreen) was used as the intervention in the study group, and AAV9-F4/80-Control-ZsGreen as the negative control. Mice were randomly divided into four groups: Sham group (normal saline, NS), I/R group (NS), I/R + AAV9-F4/80-Control group, and I/R + AAV9-F4/80-Metrnl group. After 1 week of acclimatization, mice in the latter two groups were injected with AAV9-F4/80-Metrnl or AAV9-F4/80-control virus (concentration: 1.9 × 10^12^ vg/ml; dose: 100 μl/10 μg) via the tail vein. At the same time, mice in the Sham and I/R groups received the same dose of NS solution. Four weeks after transfection, myocardial I/R surgery was performed.

### In vitro interventions and groupings

To validate the effects of Metrnl on various subtypes of macrophages, M1 macrophages were obtained by treating M0 macrophages with LPS (100 ng/ml) + IFN-γ (20 ng/ml) for 24 h(Liu et al. 2020), while M2 macrophages were generated by treating M0 macrophages with IL-4 (20 ng/ml) for 24 h (He et al. 2021). The experimental group was intervened with 200 ng/ml recombinant protein Metrnl (rMetrnl) (HY-P71617, MCE, Shanghai, China) for 24 h (Chen et al. 2023a). To simulate the effect of I/R on macrophages in vitro, BMDMs were stimulated with necrotic cardiomyocytes (NCMs) (Chen et al. 2019). Subsequently, rMetrnl and the AMPK inhibitor Compound C (100 μM) were added to investigate the underlying mechanism. (Liu et al. 2019).

To establish hypoxia/reoxygenation (H/R) model, AC16 cells were cultured in serum-free hypoglycemic medium in a hypoxic incubator containing 95% N2 and 5% CO2 for 24 h, and then released back into complete DMEM medium for 6 h under normal conditions (95% air, 5% CO2) (Lu et al. 2023b). To explore the effect of Metrnl-treated macrophages on cardiomyocytes, cells were cultured in a medium composed of macrophage conditioned medium IMDM and DMEM at a 1:1 ratio. Then, the cells were divided into five groups: Control, H/R, H/R + IMDM (Control), H/R + IMDM (Metrnl), and H/R + IMDM (Metrnl + Compound C).

### Enzyme-linked immuno sorbent assay (ELISA)

Mice were anesthetized again after 24 h of reperfusion for blood collection. Serum samples were separated from whole blood after centrifugation (3000 *g*, 4 °C) for 15 min. Serum troponin T concentration was measured by cTnT ELISA kit (ELK6207, ELK Biotechnology, China) according to the instruction. For in vitro experiments, macrophage cell supernatants were taken to detect inflammatory factors IL-1β (GEM0002, Servicebio), IL-6 (GEM0001, Servicebio), TNF-α(GEM0004, Servicebio), and anti-inflammatory factor IL-10 (GEM0003. Servicebio) levels.

### Infarct area measurement

The infarct area after MI/RI was measured by double Evans blue-triphenyltetrazolium chloride (TTC) staining. After 24-h reperfusion, mice were ventilated with a ventilator. A 2% Evans blue solution (Sigma Aldrich, USA) was slowly injected into the right ventricle, and the ascending aorta was clamped. The hearts were quickly excised, stained blue, and frozen at − 20 °C for 30 min. They were then sectioned into 4–5 segments 2 mm below the ligature, each about 1–2 mm thick, along the left ventricle longitudinal axis. After transection, the hearts were immersed in 2% TTC solution (Solarbio) for 30 min. Finally, the infarcted tissue was stained white, the surviving tissue in the ischemic area was stained red, and the nonischemic tissue was stained blue.

### Echocardiography

To compare cardiac function in each group, mice underwent echocardiography 24 h after sham or I/R surgery. Echocardiographic analysis was performed using a Vevo 2100 (Visualsonics, Toronto, Ontario, Canada). Briefly, mice were anesthetized using isoflurane (1.5% mixed with oxygen). After sufficient anesthesia was achieved, the mice were gently restrained on a temperature-controlled (37 °C) platform, and commercially available depilatory creams were used to remove hair from the ventral thorax of the mice. During the imaging acquisition, mice were continuously monitored to maintain a desirable heart rate (above 400 beats per minute). From the papillary muscle level, ventricular M-mode and B-mode echoes were recorded and processed for further analysis. Echo parameters included left ventricular ejection fraction (LVEF) and left ventricular short-axis shortening (LVFS).

### H&E and immunohistochemistry staining

Excised hearts were fixed in 4% paraformaldehyde for 24 h, then embedded in paraffin and sectioned into 5-μm slices. These sections were dehydrated in an ethanol gradient, cleared with xylene, and stained with hematoxylin–eosin (H&E). For immunohistochemistry to detect p-NF- κB expression in ischemic cardiac tissues, fixed tissues were embedded in paraffin sections, followed by antigen retrieval and endogenous peroxidase blocking. The sections were then blocked with 3% BSA. Samples were incubated overnight with a rabbit p-NF-κB antibody (3033 T, CST, Massachusetts, USA), followed by incubation with a biotin—labeled goat secondary antibody and horseradish enzyme—conjugated streptavidin ovalbumin (#SP—9000, ZSGB—BIO, Beijing, China). All samples were photographed by the EVOS FL semi-automated imaging system (Life Technologies, Carlsbad, USA).

### Immunofluorescence staining

For immunofluorescence detection of macrophages, samples were incubated with primary antibody F4/80 (GB113373, Servicebio, Wuhan, China), CD11b (GB11058, Servicebio, Wuhan, China), CD68 (ab53444, Abcam, Cambridge, UK), CD86 (CY5238, Abways, Shanghai, China), and CD206 (12-2061-82, Invitrogen, California, USA). The next day incubation with the corresponding fluorescent secondary antibodies (ab150081, ab150084, Abcam, Cambridge, UK). DAPI was used to stain the nucleus. All samples were photographed by the EVOS FL microscope and then quantitatively analyzed by Image J (NIH, Bethesda, USA).

### Flow cytometry

BMDMs were digested with Accutase (BD, California, USA) and centrifuged (300 *g*, 4 °C) for 5 min. Subsequently, cells were washed and resuspended in 100 μl of BD Pharmingen staining buffer, and BMDMs were incubated with the appropriate fluorescent antibodies for 30 min according to the reagent instructions. Finally, macrophage proportions were calculated within 30 min using a flow cytometer with CytoFlex (Beckman, Brea, USA). Data were analyzed using FLOWJO software. Antibody information: F4/80 (11-4801-81; Invitrogen, California, America), CD11b (557,667; BD Biosciences, New York, USA), CD86 (558,703; BD Biosciences, New York, USA), and CD206 (12-2061-82, Invitrogen, California, America).

### Apoptosis detection

Apoptosis was detected in sectioned tissues using the DAB-TUNEL kit method (Roche, Basel, Switzerland). After fixation with 4% paraformaldehyde, the sections were treated with 0.3% Triton X-100. Samples were then incubated with biotin- dutp or FITC-12-dUTP labeled TUNEL reaction solution. Finally, all samples were imaged with an EVOS FL microscope system and measured with Image J software.

### RT-qPCR analysis

Total RNA was extracted from cardiac tissues and cells by using TRIzol™ reagent (Invitrogen, LA, USA). Subsequently, 1 µg of RNA was reverse-transcribed into cDNA using the corresponding reagent (Takara, Tokyo, Japan) in a T100 Thermal Cycler (BioRad, USA). Target genes were amplified with TB Green solution (Takara) in CFX96 Touch (Bio-Rad, USA). Relative expression analysis was performed using the 2^−ΔΔCt^ method. Table S1 lists all the primers used in this study. All the primer species in this study are from mice.

### Western blotting

Total proteins were extracted from cells and myocardial tissue by mixing RIPA solution with PMSF (Solarbio) at a 100:1 ratio. Denatured protein samples were separated by 10% SDS-PAGE (P2012, NCM Biotech) and transferred to Millipore's PVDF membranes (Boston, USA). Membranes were blocked with 3% BSA solution (Solarbio) and incubated overnight at 4 °C with rabbit primary antibody: Metrnl (Immunoway, YT7556, Jiangsu, China; 1:1000), AMPK (AB32047, abcam, Cambridge, UK; 1:1000), p-AMPK (AF3423, Affinity, Jiangsu, China; 1:1000), BCL-2 (#3498, CST, Boston, USA; 1:1000), BAX (#2772, CST, Boston, USA; 1:1000), β-actin (AF7018, affinity, Jiangsu, China; 1:10,000), and GAPDH (GB15002-100, Servicebio, Wuhan, China; 1:1000). Then, membranes were incubated with a 1:10,000 enzyme-labeled goat secondary antibody (#ab6721, Abcam). All blots were visualized with Proteinsimple’s FluorChem FC3 system (Silicon Valley, USA) and quantified with Image J.

### Statistical analysis

All experiments were repeated at least three times. Data were statistically analyzed using IBM SPSS 22.0 software (New York, NY, USA) and expressed as means plus standard deviation (means ± SD). Comparisons of variance between two groups were performed using unpaired Student’s t-test, and differences between multiple groups were analyzed using one-way ANOVA with Tukey's multiple comparison test or Holm-Sidak test. Differences were statistically significant, *P < 0.05, **P < 0.01, ***P < 0.001, ****P < 0.0001.

## Results

### Metrnl in macrophages is downregulated after MI/RI, and overexpression of Metrnl attenuates MI/RI

**Fig. 1:**
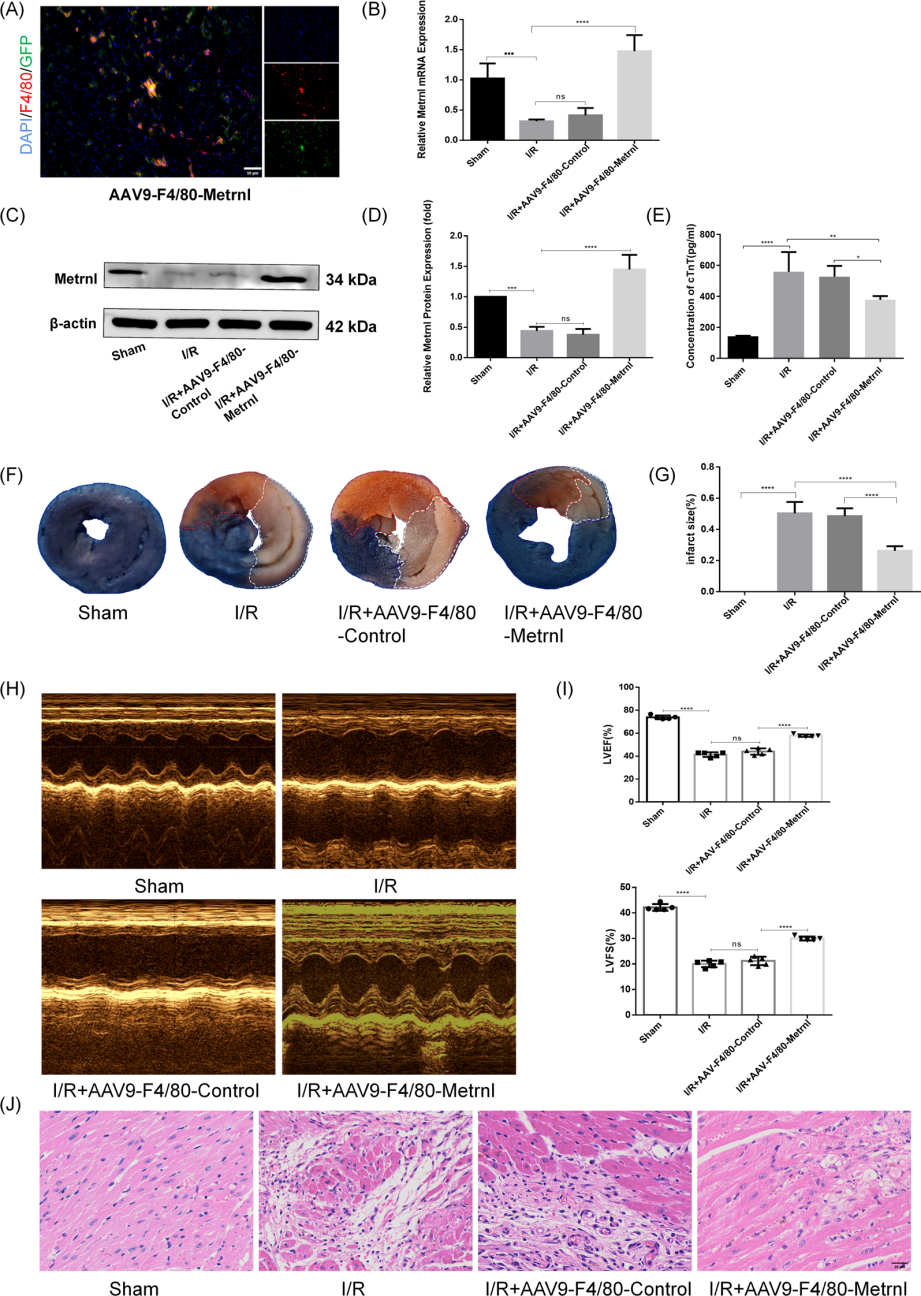
Metrnl in macrophages is downregulated after MI/RI, and overexpression of Metrnl attenuates MI/RI. **A** Immunofluorescence co-localization of F4/80 (red) and AAV9 (GFP) in mouse hearts at 4 weeks post-injection of AAV9-F4/80-Metrnl virus. Scale bar = 50 μm, magnification = 400x (n = 5). **B** qRT-PCR was employed to detect the expression of Metrnl in the infarct zone of mice in each group following I/R surgery (n = 5). **C** WB was used to compare the expression of Metrnl protein in the infarct zone of mice (n = 4). **D** Quantification of panel C.The Metrnl protein expression is the ratio of Metrnl protein to β-actin (n = 4). **E** Serum cTnT levels of mice in each group were measured by ELISA kits (n = 6). **F** Evans blue-triphenyltetrazolium chloride (TTC) staining was utilized to determine the infarct area. Area-at-risk area is red and infarct area is white (n = 5). **G** Infarct area (%) was expressed as the percentage of white area to total area (n = 5). **H** Echocardiography was performed to assess cardiac function. **I** Left ventricular ejection fraction (LVEF) and left ventricular shortening fraction (LVFS) were compared between groups of mice (n = 5). **J** H&E staining revealed the morphological changes of myocardial tissue in each group of mice (n = 6). Scale bar = 20 μm, magnification = 400x. Data are presented as means ± SD, *ns* not statistically significant; *P < 0.05, **P < 0.01, ***P < 0.001, ****P < 0.0001

To investigate the effect of Metrnl on MI/RI, an adeno-associated virus containing the F4/80 promoter (AAV9-F4/80) was used to overexpress Metrnl in cardiac macrophages. Immunofluorescence staining revealed co-localization of GFP carried by AAV9 with F4/80(Red), which indicated that AAV9-F4/80-Metrnl successfully targeted the cardiac macrophages (Fig. 1A). RT-qPCR experiments demonstrated that the expression of Metrnl in the infarcted myocardial region was significantly decreased after MI/RI, whereas the expression of Metrnl was significantly increased after AAV9-F4/80-Metrnl transfection (Fig. 1B). Consistent with the results of qRT-PCR experiments, WB results showed that Metrnl expression was significantly elevated after AAV9-F4/80-Metrnl transfection (Fig. 1C, D). The above experimental results fully proved that the expression of Metrnl on macrophages decreased after MI/RI, and AAV9-F4/80-Metrnl successfully overexpressed Metrnl in cardiac macrophages. ELISA results indicated that the serum troponin T (cTnT) levels in the I/R + AAV9-F4/80-Metrnl group were significantly decreased compared with the I/R group and the I/R + AAV9-F4/80-Control group (Fig. 1E). Evans blue-TTC staining revealed that the infarct area in the I/R + AAV9-F4/80-Control group was not significantly different from that in the I/R group, whereas the infarct area in the I/R + AAV9-F4/80-Metrnl group was significantly reduced (Fig. 1F, G). Echocardiographic results showed that LVEF and LVFS were significantly lower in the I/R group and I/R + AAV9-F4/80-Control group compared with the Sham group, whereas LVEF and LVFS were significantly higher in the I/R + AAV9-F4/80-Metrnl group compared with the above two groups (Fig. 1H, I). H&E staining demonstrated that myocardial edema and inflammatory infiltration were significantly reduced in the I/R + AAV9-F4/80-Metrnl group compared with the I/R and I/R + AAV9-F4/80-Control groups (Fig. 1J). These results suggest that Metrnl expression is downregulated on macrophages after MI/RI and its overexpression may attenuate MI/RI.

### Overexpression of Metrnl in macrophages attenuates I/R-induced inflammatory response and cardiomyocyte apoptosis

**Fig. 2:**
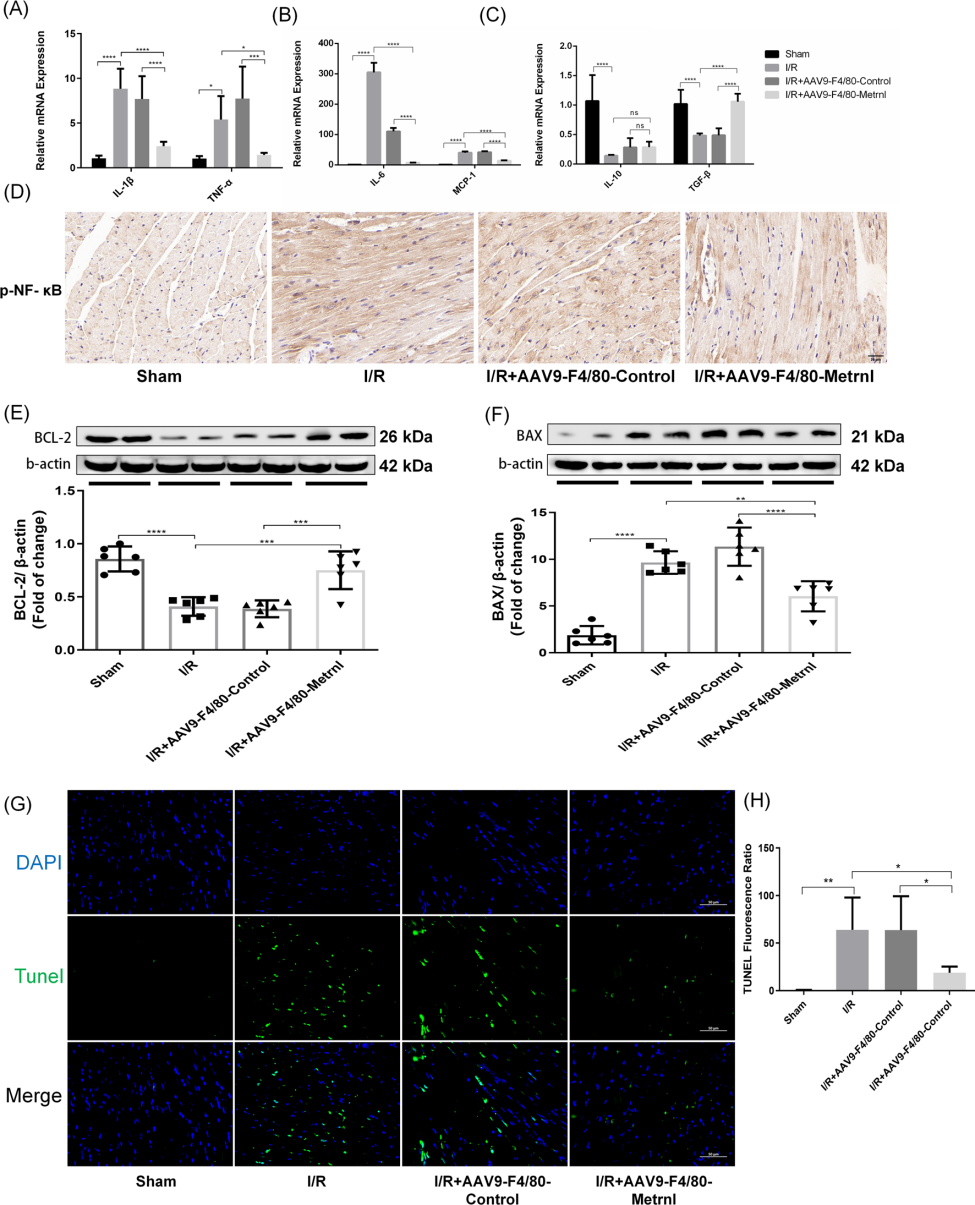
Overexpression of Metrnl in macrophages attenuates I/R-induced inflammatory response and cardiomyocyte apoptosis. **A**–**C** qRT-PCR was performed to detect the expression levels of inflammatory factors IL-1β, TNF-α, IL-6, MCP-1 and anti-inflammatory factors IL-10 and TGF-β in each group, respectively (n = 5). **D** Immunohistochemistry was used to compare the expression levels of p-NF-κB in each group (n = 6). Scale bar = 20 μm, magnification = 400x. **E** WB was employed to detect the expression of anti-apoptotic protein BCL-2 and its quantitative comparison (n = 6). **F** WB was used to detect the expression of apoptotic protein Bax and its quantitative comparison (n = 6). **G** TUNEL staining was conducted to compare the apoptosis rate of each group (n = 6). Scale bar = 50 μm, magnification = 400x. **H** Quantification of panel G. The apoptosis rate (%) was calculated as the number of nuclei stained by green fluorescence divided by the number of all nuclei (n = 6). Data are presented as means ± SD, *ns* not statistically significant; *P < 0.05, **P < 0.01, ***P < 0.001, ****P < 0.0001

Apoptosis and macrophage-induced inflammatory responses play pivotal roles in the progression of MI/RI. To further investigate the impact of Metrnl on inflammatory responses and cell apoptosis, we conducted a series of experiments. RT-qPCR results revealed that, compared with the Sham group, expression levels of inflammatory cytokines such as IL-1β, TNF-α, IL-6, and MCP-1 were significantly elevated in both the I/R and I/R + AAV9-F4/80-Control groups, while the expression of anti-inflammatory cytokines IL-10 and TGF-β was markedly decreased. In contrast, the I/R + AAV9-F4/80-Metrnl group exhibited significantly reduced expression of inflammatory cytokines and slightly increased expression of anti-inflammatory cytokines compared to the above two groups (Fig. 2A–C). Immunohistochemical analysis demonstrated that phosphorylated NF-κB (p-NF-κB), closely associated with inflammatory responses, was notably upregulated after MI/RI, while overexpression of Metrnl in cardiac macrophages downregulated p-NF-κB expression levels (Fig. 2D). These findings suggest that Metrnl can mitigate I/R-induced inflammatory responses. WB results indicated that the anti-apoptotic protein BCL-2 was downregulated in the I/R and I/R + AAV9-F4/80-Control groups compared with the Sham group, whereas overexpression of Metrnl upregulated BCL-2 expression (Fig. 2E). Conversely, the apoptotic protein BAX was upregulated after MI/RI surgery, but Metrnl overexpression downregulated BAX expression (Fig. 2F). TUNEL assays showed that overexpression of Metrnl in cardiac macrophages significantly decreased the apoptosis rate in the cardiac infarction area compared with the I/R and I/R + AAV9-F4/80-Control groups (Fig. 2G, H). These results demonstrate that Metrnl can alleviate I/R-induced cell apoptosis.

### Metrnl overexpression promotes macrophage polarization from the M1 to the M2 subtype

**Fig. 3:**
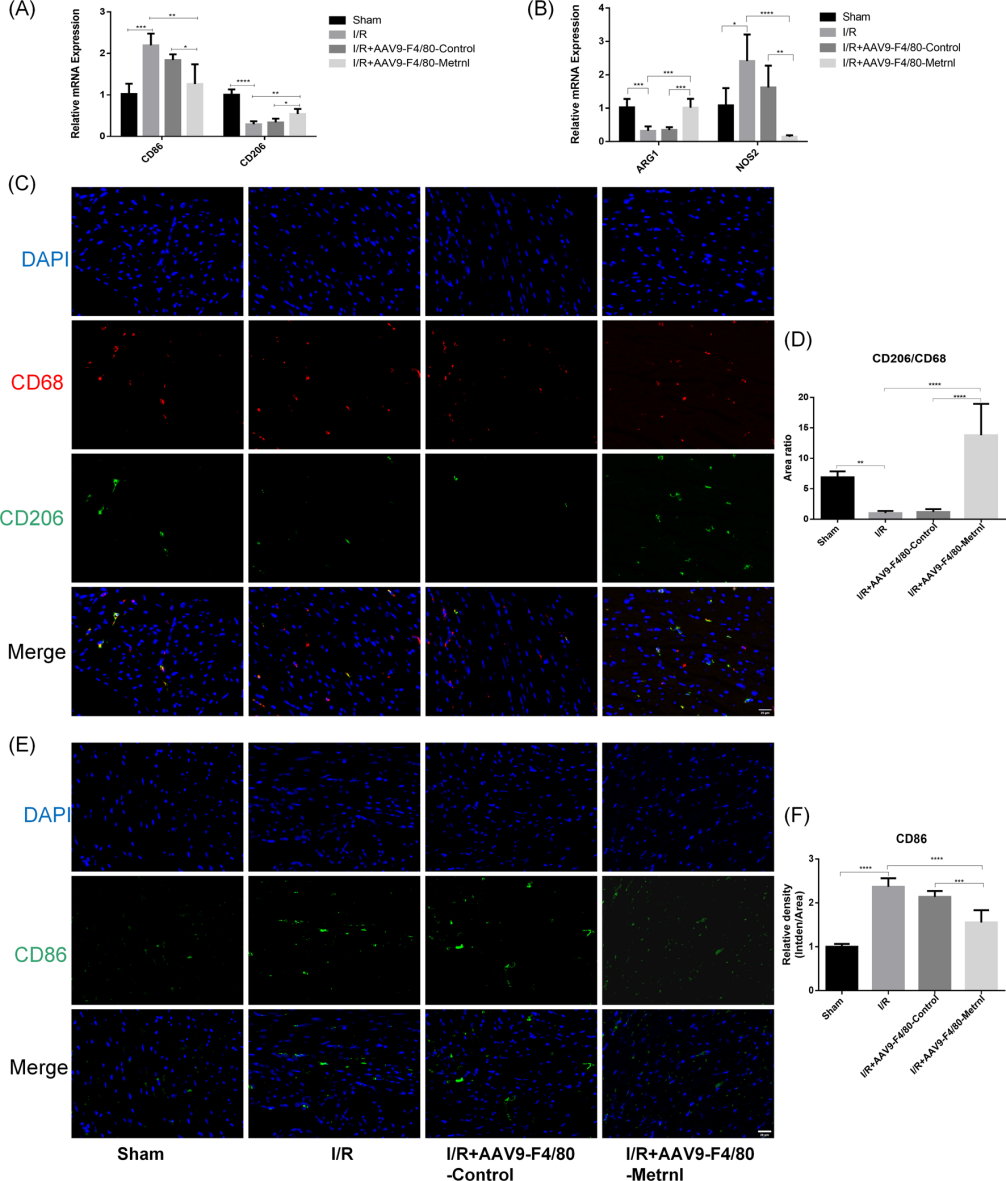
Metrnl overexpression promotes macrophage polarization from the M1 to the M2 subtype. **A**, **B** qRT-PCR was performed to detect CD86,CD206,ARG1,NOS2 mRNA levels in the infarct zone of each group (n = 5). **C** Immunofluorescence staining was performed to determine the expression of CD206 (green). Scale bar = 20 μm, magnification = 400x (n = 6). **D** Quantification of panel C. CD206/CD68 indicates the percentage of CD206 fluorescence area to CD68 fluorescence area (n = 6). **E** Immunofluorescence staining was performed to determine the expression of CD86 (green). Scale bar = 20 μm, magnification = 400x (n = 6). **F** Quantification of panel E. CD86 mean fluorescence intensity indicates the percentage of CD86 gray value and fluorescence area (n = 6). Data are presented as means ± SD, *ns* not statistically significant; *P < 0.05, **P < 0.01, ***P < 0.001, ****P < 0.0001

Given the pro-inflammatory role of M1 macrophages and the anti-inflammatory role of M2 macrophages, we further investigated whether Metrnl can regulate macrophage polarization from the M1 to the M2 phenotype to attenuate MI/RI. qRT-PCR results demonstrated that following MI/RI surgery, the expression of M1 macrophage markers such as CD86 and NOS2 increased in the infarcted area, while the expression of M2 macrophage markers including CD206 and ARG1 decreased. Overexpression of Metrnl in cardiac macrophages resulted in a significant reduction in CD86 and NOS2 expression and an increase in CD206 and ARG1 expression (Fig. 3A, B). Similarly, immunofluorescence staining revealed a significant decrease in CD206 expression in the infarcted area of the heart in both the I/R group and the I/R + AAV9-F4/80-Control group compared to the Sham group, whereas CD206 expression was upregulated in the I/R + AAV9-F4/80-Metrnl group (Fig. 3C, D). Conversely, the expression of CD86 significantly increased after MI/RI surgery, but overexpression of Metrnl in cardiac macrophages led to a reduction in CD86 expression in the infarcted area (Fig. 3E, F). These findings suggest that overexpression of Metrnl promotes the polarization of macrophages from the M1 to the M2 phenotype following MI/RI surgery.

### Effect of Metrnl on macrophage polarization in vitro

**Fig. 4:**
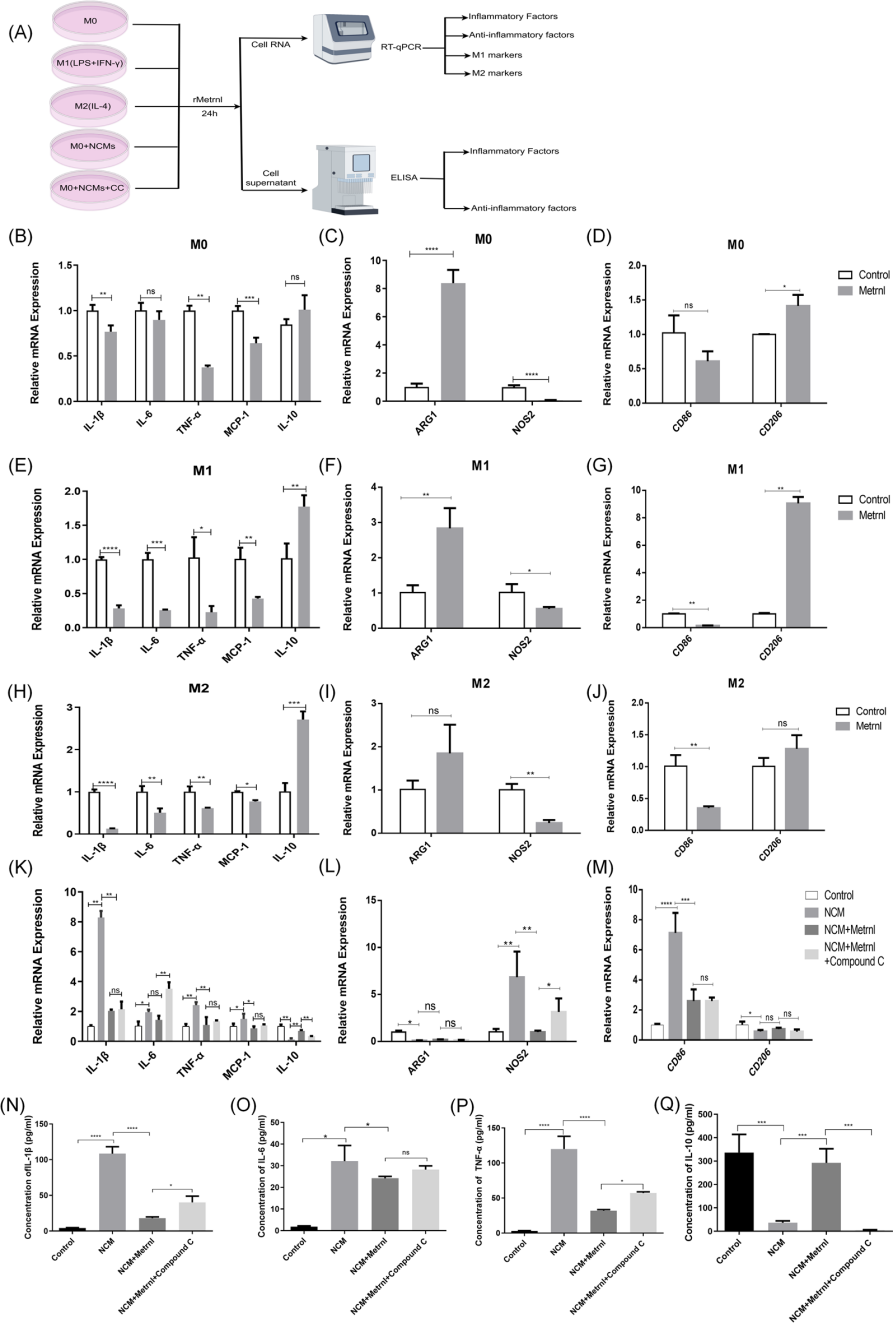
Effect of Metrnl on macrophage polarization in vitro. **A** Diagram of the experimental protocol for the effect of Metrnl on macrophage polarization. **B**–**D** qRT-PCR compared mRNA levels of inflammatory and anti-inflammatory factors in bone marrow-derived macrophages (BMDMs) and after Metrnl intervention. The recombinant protein Metrnl intervention was 200 ng/mL for 24 h, as below. **E**–**G** qRT-PCR detected mRNA levels of inflammatory and anti-inflammatory factors in M1-type macrophages and after Metrnl intervention. M1-type macrophages were obtained by treating BMDMs with LPS (100 ng/ml) and IFN-γ (20 ng/ml) for 24 h. **H**–**J** qRT-PCR for mRNA levels of inflammatory and anti-inflammatory factors in M2-type macrophages and after Metrnl intervention. M2-type macrophages were obtained by treating BMDMs with IL-4 (20 ng/ml) for 24 h. (K-M) qRT-PCR detected mRNA levels of inflammatory and anti-inflammatory factors in each group. (N-Q) ELISA kits detected levels of inflammatory factors IL-1β, IL-6, TNF-α and anti-inflammatory factor IL-10 in macrophage supernatants. “rMetrnl”: recombinant protein Metrnl. NCMs: necrotic cardiomyocytes. *CC* Compound C. Control group: BMDMs. NCM group: stimulated with NCMs for 24 h. NCM + Metrnl group: intervened with NCMs and recombinant protein Metrnl. NCM + Metrnl + Compound C group: stimulated for 2 h by adding Compound C (100 μM/L) to NCM + Metrnl group. All tests were repeated independently at least three times. Data are presented as means ± SD, ns: not statistically significant; *P < 0.05, **P < 0.01, ***P < 0.001, ****P < 0.0001

To further determine the effect of Metrnl on macrophage polarization, BMDMs were extracted and identified by immunofluorescence staining (Figure S1). We used the recombinant protein Metrnl (rMetrnl) to intervene in M0, M1, and M2 macrophages, as well as macrophages stimulated by NCMs respectively. Then, we detected the inflammatory factors and anti-inflammatory factors, as well as the polarization status of macrophages by RT-qPCR and ELISA respectively (Fig. 4A). qRT-PCR results showed that based on M0 macrophages, compared with the Control group, Metrnl led to a decrease in the expression of inflammatory factors including IL-1β, TNF-α, and MCP-1, and an slightly increase (ns) in the expression of anti-inflammatory factor IL-10 (Fig. 4B). At the same time, NOS2 were significantly downregulated, while CD206 and ARG1 were significantly upregulated (Fig. 4C, D). Moreover, based on M1 macrophages, Metrnl also significantly attenuated the expression of the above inflammatory factors and increased the expression of IL-10 (Fig. 4E), while reducing the expression levels of CD86 and NOS2 and increasing the expression of CD206 and ARG1 (Fig. 4F, G). Similar results were obtained on the basis of M2 macrophages. Metrnl decreased the level of inflammatory factors, increased the level of anti-inflammatory factors, weakened M1 polarization, and marginally enhanced M2 polarization (Fig. 4H–J).

To simulate the effects of the microenvironment on macrophages after MI/RI, we stimulated macrophages with NCMs. This stimulation significantly increased proinflammatory cytokines and decreased IL-10 levels. CD86 and NOS2 were substantially upregulated, whereas CD206 and ARG1 were significantly downregulated, suggesting that macrophages underwent a proinflammatory transition. When intervening with rMetrnl, the changes of CD206 and ARG1 were not obvious (ns), but an upward trend was still observable. Notably, Metrnl effectively decreased the expression of pro-inflammatory cytokines IL-1β, TNF-α and MCP-1, increased IL-10, and inhibited CD86 and NOS2, suggesting an anti-inflammatory effect. The anti-inflammatory effect of Metrnl was not completely reversed by the AMPK inhibitor Compound C under NCM stimulation. Although the increases in IL-1β, TNF-α and MCP-1 were not significant, Compound C showed a reversing tendency, suggesting that Metrnl may act via the AMPK pathway. (Fig. 4K–M).

Similarly, the detection results of macrophage supernatant by ELISA showed that compared with the Control group, the NCM group showed a significant increase in the expression of inflammatory cytokines, and a decrease in the expression of IL-10. In contrast, the NCM + Metrnl group had decreased levels of inflammatory cytokines and increased expression of IL-10, and these effects were inhibited by Compound C (Fig. 4N–Q). These findings suggest that Metrnl may promote macrophage polarization from M1 to M2, inhibit the release of inflammatory cytokines, and enhance the release of anti-inflammatory cytokines through the AMPK pathway.

### Metrnl attenuates H/R-induced cardiomyocyte apoptosis via AMPK-mediated M1-to-M2 macrophage polarization

**Fig. 5:**
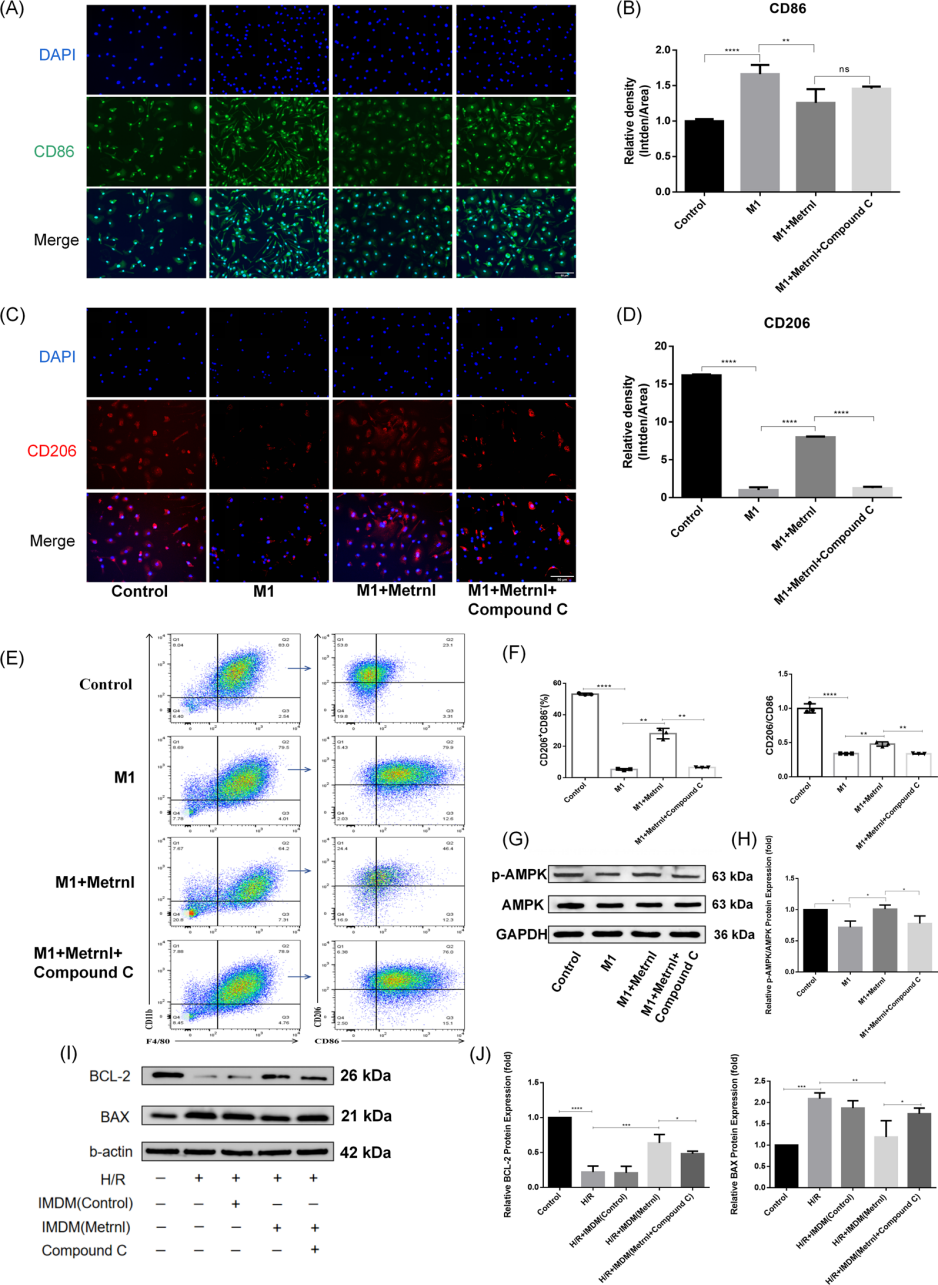
Metrnl attenuates H/R-induced cardiomyocyte apoptosis via AMPK-mediated M1-to-M2 macrophage polarization. **A** Immunofluorescence staining detected CD86 (green) expression in each group. Scale bar = 50 μm, magnification = 200 × . **B** Quantification of panel A. Mean fluorescence intensity of CD86 = CD86 gray value/fluorescence area. **C** Immunofluorescence staining detected CD206 (red) expression in four groups. Scale bar = 50 μm, magnification = 200 × . **D** Quantification of panel C. CD206 mean fluorescence intensity = CD206 gray value/fluorescence area. **E** Flow cytometry detected macrophage polarization in four groups. **F** Quantification of panel E. **G** WB experiments verified p-AMPK and AMPK protein expression in four groups. **H** Quantification of panel G. Relative expression of p-AMPK = p-AMPK/AMPK. **I** WB detected BCL-2 and BAX protein expression in AC16 cells in each group. **J** Quantification of panel L. Relative expression of BCL-2 = BCL-2/GAPDH; relative expression of BAX = BAX/GAPDH. All tests were repeated independently at least three times. Data are presented as means ± SD, *ns* not statistically significant; *P < 0.05, **P < 0.01, ***P < 0.001, ****P < 0.0001

To further explore through which pathway Metrnl regulates the polarization of M1 macrophages to M2 to improve myocardial injury, we first used LPS and IFN-γ to polarize BMDMs to the M1 subtype. The immunofluorescence staining results showed that the fluorescence intensity of CD86 was enhanced in the M1 group, weakened in the M1 + Metrnl group, and slightly enhanced again after adding Compound C (Fig. 5A, B). In contrast, the fluorescence intensity of CD206 was weakened in the M1 group, and enhanced in the M1 + Metrnl group, and this effect was reversed by Compound C (Fig. 5C, D). Through flow cytometry analysis, compared with the M1 group, the proportion of CD206^+^CD86^−^ macrophage population and the ratio of CD206^+^ to CD86^+^ increased in the M1 + Metrnl group, while Compound C reversed these increases (Fig. 5E, F). WB results showed that compared with the Control group, the ratio of P-AMPK to AMPK decreased in the M1 group, while Metrnl increased the ratio of P-AMPK to AMPK, and Compound C reversed this effect (Fig. 5G, H). This indicates that AMPK activity is inhibited in M1 macrophages, and Metrnl may promote the polarization of macrophages from M1 to M2 by activating AMPK. Macrophage supernatant IMDM was used as the conditioned medium to co-culture with AC16 cells and then perform H/R intervention. WB experiments showed that compared with the H/R group and the H/R + IMDM (Control) group, BAX was decreased and BCL-2 was increased in the H/R + IMDM (Metrnl) group. However, IMDM (Metrnl + Compound C) increased the expression of BAX and decreased the expression of BCL-2 (Fig. 5I, J). The above experimental results indicate that Metrnl may regulate the polarization of M1 macrophages to M2 type by activating the AMPK pathway, thereby attenuating the H/R-induced apoptosis of AC16 cells.

## Discussion

In this study, we identified Metrnl as a novel regulatory factor with the potential to ameliorate MI/RI. The findings revealed a significant downregulation of Metrnl expression in cardiac macrophages following MI/RI. Furthermore, Metrnl exhibited cardioprotective properties. Specifically, the overexpression of Metrnl in cardiac macrophages reduced in infarct size, decreased inflammatory infiltration and cardiomyocyte apoptosis, and enhanced recovery of cardiac function post-MI/RI may be that Metrnl induces M2 macrophage polarization through activation of AMPK phosphorylation. In conclusion, this study further elucidates the critical role of Metrnl in the pathophysiological processes of ischemic heart disease.

Metrnl triggers various signaling pathways in adipocytes, macrophages, myocytes, and cardiomyocytes, which are crucial for insulin sensitivity, reducing inflammation, aiding muscle regeneration, and protecting the heart (Miao et al. 2020). This characteristic implies that Metrnl could be an effective target for treating acute inflammatory diseases like MI/RI. It has been demonstrated that Metrnl expression decreases in an in vitro MI/RI model, and its overexpression mitigates apoptotic injury in cardiomyocytes (Xu et al. 2020). Metrnl is highly expressed in cardiac tissues, where it plays a key role in cardiac pathophysiology by preventing cardiac hypertrophy and fibrosis and restoring normal myocardial function (Rupérez, et al. 2021). Additionally, Metrnl improves diabetic cardiomyopathy and reduces heart cell damage by inhibiting cGAS/STING signaling through autophagy (Lu et al. 2023a), but also attenuates adriamycin-induced cardiotoxicity via activation of the cAMP/PKA/SIRT1 pathway (Hu et al. 2020). In the current study, we observed reduced Metrnl levels on myocardial macrophages after MI/RI. In addition, overexpressing Metrnl in macrophages exhibited significant improvement in cardiac function, as indicated by decreased cTnT expression, reduced myocardial infarct size, and increased LVEF and LVFS. Notably, a recent study by Li et al. demonstrated that Metrnl expression was significantly elevated in infarcted areas of mice and tissues of patients with AMI (Li et al. 2023). This contradictory result may be attributed to following factors. First, the pathogenesis of AMI is different from that of MI/RI, which is accompanied by oxidative stress, calcium overload, complement activation, and inflammatory burst in addition to massive cardiomyocyte necrosis (Algoet et al. 2023). These pathogenic differences might account for the divergent Metrnl trends between MI/RI and AMI. Second, our time for MI/RI detection was early after reperfusion (within 24 h), when the acute inflammatory response predominates and Metrnl may be rapidly consumed (Francisco and Re 2023). However, Li et al. may have detected it at a more advanced stage (repair), when the tissue repair requires the involvement of Metrnl, leading to the upregulation of expression. Furthermore, Li et al.’s study centered on whole infarcted tissue, whereas our current work focuses more on infarct area macrophages. Early reperfusion is dominated by M1 macrophages (Shen et al. 2024), while Metrnl is chiefly secreted by M2 macrophages (Li et al. 2023), which may have led to a decrease in the expression of Metrnl on macrophages. Finally, different studies may have used different detection techniques and antibodies, and these factors may affect the accurate assessment of Metrnl expression levels. In the future, further experimental methods such as flow cytometry, comparison after constructing MI/RI and AMI models, multi-omics joint analysis and clinical sample validation can be used to clarify the reasons for the differences in Metrnl expression.

It is well established that MI/RI is accompanied by substantial inflammatory infiltration. To investigate Metrnl’s effect on MI/RI-induced inflammation, experiments were performed at both pathological and molecular levels. H&E staining showed that Metrnl reduced neutrophil infiltration and cell edema post-I/R. Additionally, Metrnl decreased inflammatory factors IL-1β, IL-6, TNF-α, and MCP-1, while increasing anti-inflammatory factors IL-10 and TGF-β. Immunohistochemistry confirmed that Metrnl overexpression significantly decreased phosphorylated NF-κB levels. Collectively, our study shows that Metrnl reduces MI/RI-induced inflammation, supporting previous findings of its anti-inflammatory effects, such as in allergic asthma (Gao et al. 2022) and LPS-induced muscle inflammation (Jung et al. 2018). The mechanisms by which Metrnl modulates inflammatory responses still require in-depth exploration.

Macrophages play a key role in the MI/RI process, and the direction of their polarization has a profound effect on MI/RI development (Fan et al. 2019). Metrnl promotes adipose tissue M2-type macrophage polarization and enhances thermogenesis and anti-inflammation, whereas blocking Metrnl significantly attenuates alternative macrophage activation triggered by chronic cold exposure (Rao et al. 2014). Our study found that cardiac macrophages overexpressing Metrnl inhibited M1-type polarization and promoted M2-type polarization. Based on M0 and M1 macrophages, Metrnl inhibited M1 polarization and promoted M2 polarization, decreased the release of inflammatory factors such as IL-1β, IL-6, and TNF-α, and increased the secretion of IL-10. This is consistent with previous studies that Metrnl not only promotes M2 macrophage activation in the high-fat obesity setting (Rao et al. 2014), but also exerts a similar effect in the diabetic setting (Song et al. 2023). Regrettably, Metrnl did not show a clear trend towards pro-M2 macrophage polarization on an M2 macrophage basis. From the cellular point of view, the polarization state of M2 macrophages is relatively stable, and their gene expression and function are relatively fixed (Chen et al. 2023b). Alternatively, they are heterogeneous and some cells may be insensitive to Metrnl (Kumar Jha et al. 2024). From an experimental point of view, limited sample size, poor sensitivity of the assay and subtle differences in culture conditions may affect the results. Although there was no statistically significant difference between ARG1 and CD206 elevation, the trend of elevation implied that Metrnl might maintain M2 status and enhance anti-inflammatory function. Further studies are needed to examine the ways in which Metrnl promotes M2 polarization to reduce tissue inflammation.

AMPK, a key energy receptor, has emerged as a potential pharmacotherapeutic target in MI/RI, with its activation effectively alleviating myocardial inflammatory infiltration and cardiomyocyte apoptosis (Qin et al. 2023). Previous studies show that AMPK activity is suppressed in M1 macrophages but is restored when they shift to the M2 type (Xu et al. 2021). Consistent with these findings, our study revealed that AMPK activity was diminished in M1 macrophages, while Metrnl intervention significantly enhanced AMPK phosphorylation levels, indicating Metrnl's capacity to activate AMPK. Moreover, with the application of the AMPK inhibitor Compound C, macrophage polarization towards M1 was augmented and M2 polarization was attenuated, accompanied by an increased release of inflammatory factors and a decreased release of anti-inflammatory factors. Notably, our study showed that under NCM—stimulated macrophage conditions, Metrnl failed to significantly promote M2 polarization, whereas the inhibition of M1 markers was highly synchronized with the reduction of pro—inflammatory factors (Fig. 4K–M). This could be attributed to the overwhelming nature of the NCM-induced inflammatory microenvironment. The complex network of cytokines and damage-associated molecular patterns (DAMPs) released by NCM may activate M1 polarization signaling and interfere with the downstream signaling of AMPK that is crucial for M2 polarization (Biemmi et al. 2020; Liu et al. 2022). However, the results from our flow cytometry and immunofluorescence staining experiments showed that Metrnl not only inhibited M1 polarization but also promoted M2 polarization by activating AMPK (Fig. 5A–F). In any case, the overall effect of Metrnl on macrophage polarization status implies a potential link to activation of AMPK to promote M2 polarization.

Previous studies have found that Metrnl lessened apoptosis and inflammation by blocking the PI3K/Akt/NF-κB pathway and protected pancreatic β-cells from apoptosis through the WNT/β-catenin pathway (Liu et al. 2023; Hu et al. 2021). Similarly, our study demonstrated that Metrnl overexpression in macrophages attenuated MI/RI-induced cardiomyocyte apoptosis. To further validate the role of Metrnl on I/R injury in vitro, we used H/R to simulate the MI/RI model. In the ischemic phase, hypoxia (95% N₂ + 5% CO₂) combined with serum-free low-glucose medium simulated the disruption of oxygen and nutrient supply due to the interruption of coronary blood flow, forcing cardiomyocytes to rely on anaerobic glycolysis for energy supply, which triggers ATP depletion, lactate buildup, intracellular acidosis, and damage exacerbated by an imbalance in calcium homeostasis and a decrease in mitochondrial membrane potential (Wu et al. 2022). During the reoxygenation phase (95% air + 5% CO₂ in complete medium), the restoration of oxygen and glucose supplies triggers an outburst of ROS in the mitochondrial respiratory chain, calcium overload, and an inflammatory cascade, which exacerbates the oxidative stress and cell-death mechanisms associated with reperfusion injury (Yang et al. 2021). This model effectively simulated the “double-strike” effect of MI/RI, the synergistic effect of energy crisis during ischemia and oxidative damage during reperfusion. Expectedly, Metrnl-treated macrophage supernatants notably lessened H/R-induced apoptosis, and Compound C weakened Metrnl's anti-apoptotic effect, indicating that Metrnl may promote M2 macrophage polarization through activation of AMPK thereby reducing cardiomyocyte apoptosis. Based on these findings, Metrnl may serve as a promising agent target due to its role in preserving a healthy cardiac microenvironment, ultimately mitigating inflammatory damage and apoptosis following MI/RI.

There remain several limitations to this study. First, the function of Metrnl has not been reverse-validated, which could be verified in the future by cardiac macrophage-specific knockout mice. Second, the mechanism of action is not detailed enough, and the direct molecules engaged in Metrnl's modulation of M2 macrophage polarization can be further explored by protein interactions and pull-down experiments. Finally, it lacks further clinical validation, and myocardial and blood samples from AMI patients undergoing emergency PCI can be collected to analyze the relationship between changes in Metrnl expression and patient prognosis. Nevertheless, this study still revealed the important role of Metrnl in MI/RI and its regulatory mechanism on macrophage polarization.

## Conclusion

**Fig. 6:**
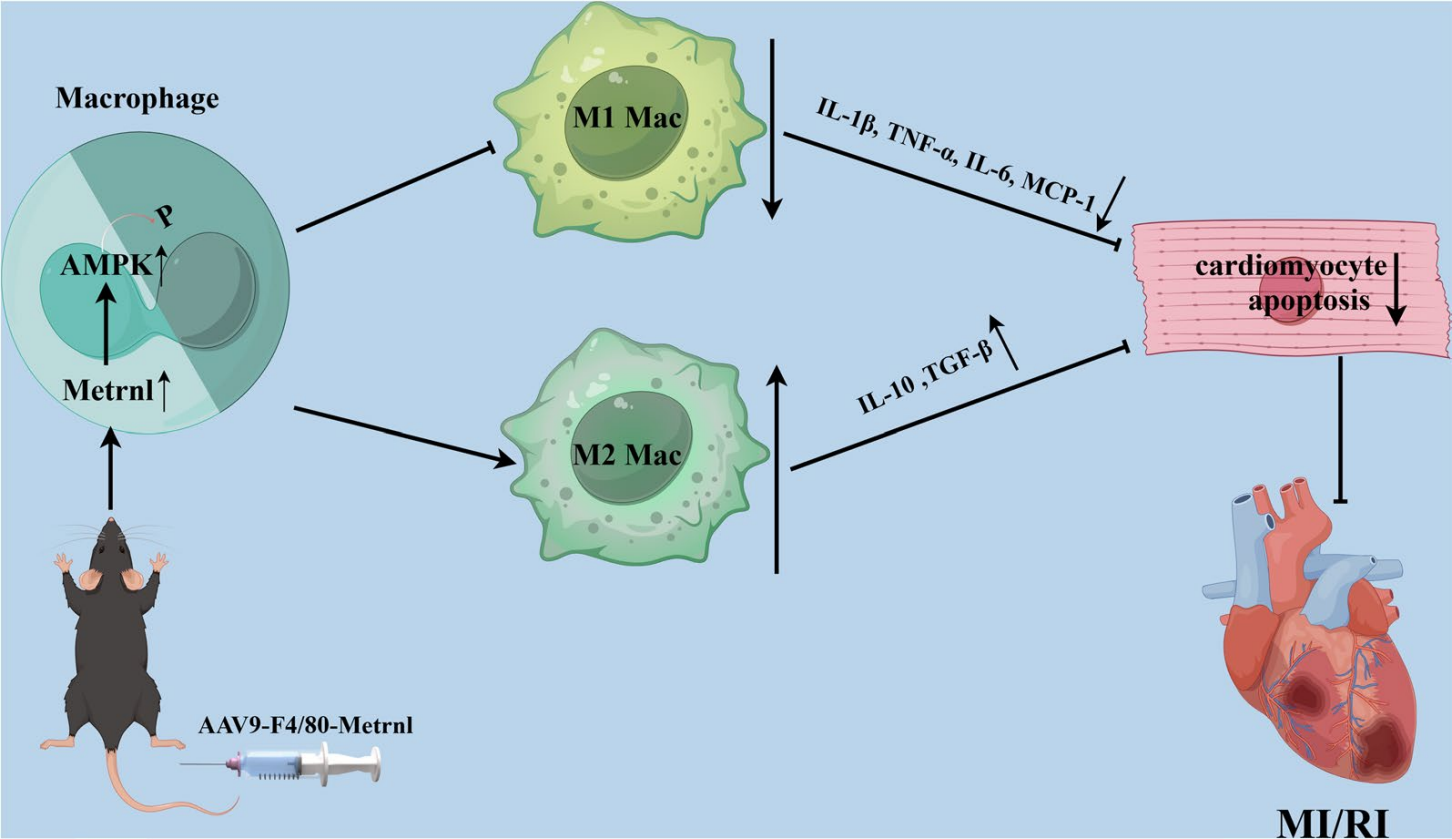
Molecular mechanism diagram. It revealed that Metrnl alleviated MI/RI by activating AMPK-mediated M2 macrophage polarization to attenuate inflammatory response and cardiomyocyte apoptosis

In conclusion, we found that Metrnl might alleviate MI/RI by activating AMPK-mediated M2 macrophage polarization to attenuate inflammatory response and cardiomyocyte apoptosis (Fig. 6). This study highlights the therapeutic potential of Metrnl in MI/RI, and identifies it as a promising target for the treatment of ischemic heart disease.

## Supplementary Information

Supplementary material 1.

Supplementary material 2.

## Data Availability

No datasets were generated or analysed during the current study.
