# Supplementary figures and images for: Metrnl ameliorates myocardial ischemia–reperfusion injury by activating AMPK-mediated M2 macrophage polarization
Source: Mol Med. 2025 Mar 13;31:98. doi: 10.1186/s10020-025-01150-4 (PMC11907862; doi:10.1186/s10020-025-01150-4)

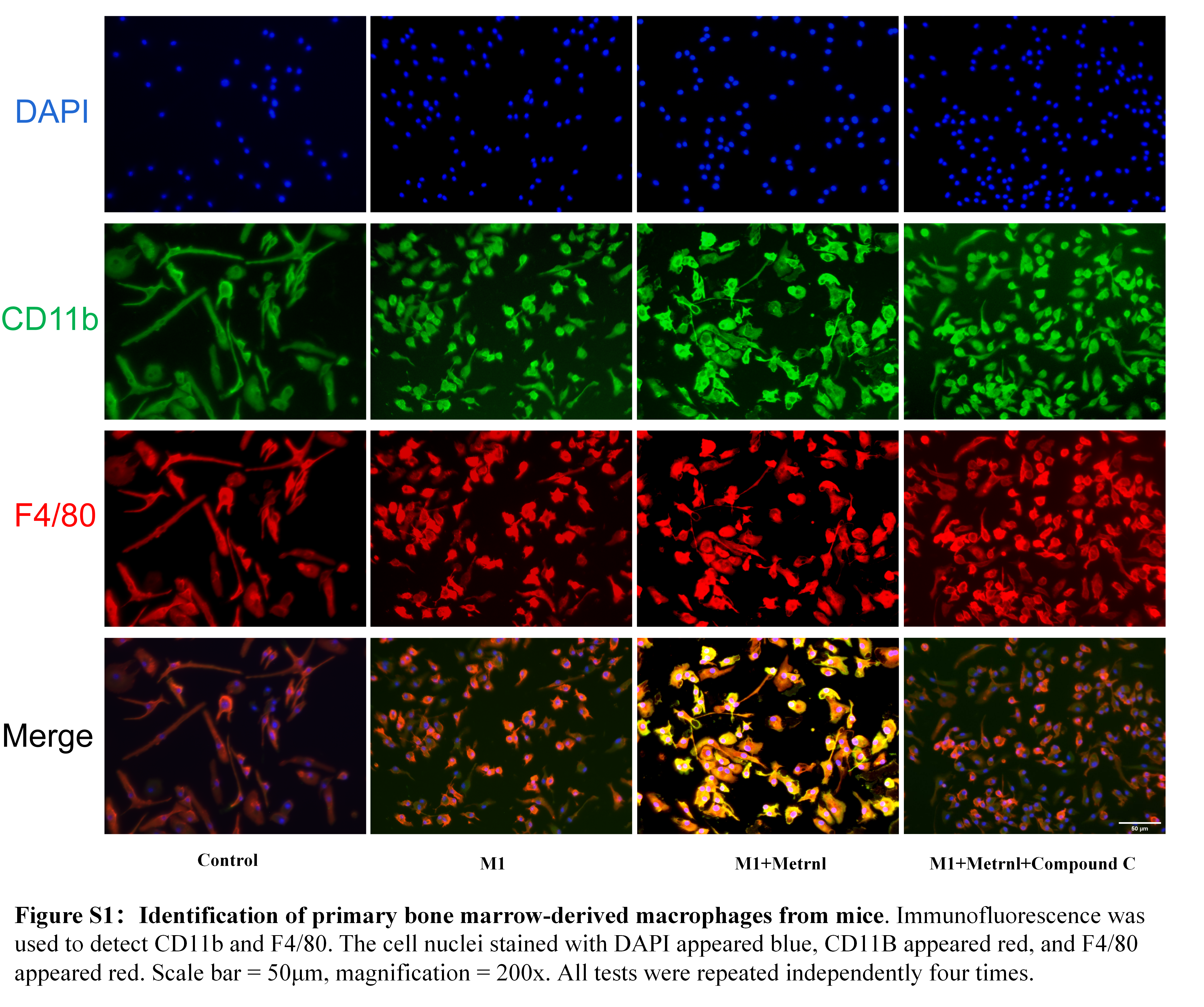

Supplement: Supplementary file 1 — Supplementary material 1. [file 10020_2025_1150_MOESM1_ESM.tif]
