# Supplementary material for: Metrnl ameliorates myocardial ischemia–reperfusion injury by activating AMPK-mediated M2 macrophage polarization
Source: Mol Med. 2025 Mar 13;31:98. doi: 10.1186/s10020-025-01150-4 (PMC11907862; doi:10.1186/s10020-025-01150-4)
Supplement: Supplementary file 2 — Supplementary material 2. [file 10020_2025_1150_MOESM2_ESM.pdf]

Supplementary materials

Table S1： All mouse primer sequences

Gene	Primer sequence
Metrnl	Forward:GCTGCTGTTGCTGCTACTACTG
	Reverse:TCCTTGCTGCGTGCCTCTC
IL-1b	Forward:CACTACAGGCTCCGAGATGAACAAC
	Reverse:TGTCGTTGCTTGGTTCTCCTTGTAC
IL-6	Forward: CTTCTTGGGACTGATGCTGGTGAC
	Reverse:TCTGTTGGGAGTGGTATCCTCTGTG
TNF-a	Forward::CGCTCTTCTGTCTACTGAACTTCGG
	Reverse:GTGGTTTGTGAGTGTGAGGGTCTG
MCP-1	Forward: CACTCACCTGCTGCTACTCATTCAC
	Reverse:CTTCTTTGGGACACCTGCTGCTG
IL-10	Forward:TCCCTGGGTGAGAAGCTGAAGAC
	Reverse： CACCTGCTCCACTGCCTTGC
TGF-b	Forward:ACCGCAACAACGCCATCTATGAG
	Reverse:GGCACTGCTTCCCGAATGTCTG
CD86	Forward:ACGGAGTCAATGAAGATTTCCT
	Reverse:GATTCGGCTTCTTGTGACATAC
CD206	Forward:CCTATGAAAATTGGGCTTACGG
	Reverse:CTGACAAATCCAGTTGTTGAGG
Arg1	Forward:CATATCTGCCAAAGACATCGTG
	Reverse:GACATCAAAGCTCAGGTGAATC
Nos2	Forward:ACTCAGCCAAGCCCTCACCTAC
	Reverse:TCCAATCTCTGCCTATCCGTCTCG
b-actin	Forward:CTACCTCATGAAGATCCTGACC
	Reverse:CACAGCTTCTCTTTGATGTCAC
GAPDH	Forward:GGCAAATTCAACGGCACAGTCAAG
	Reverse:TCGCTCCTGGAAGATGGTGATGG
